# Encapsulation of Salmon Peptides in Marine Liposomes: Physico-Chemical Properties, Antiradical Activities and Biocompatibility Assays

**DOI:** 10.3390/md20040249

**Published:** 2022-03-31

**Authors:** Amine Hanachi, Arnaud Bianchi, Cyril J. F. Kahn, Emilie Velot, Elmira Arab-Tehrany, Céline Cakir-Kiefer, Michel Linder

**Affiliations:** 1 Université de Lorraine, LIBio, F-54000 Nancy, France; am.hanachi@gmail.com (A.H.); cyril.kahn@univ-lorraine.fr (C.J.F.K.); elmira.arab-tehrany@univ-lorraine.fr (E.A.-T.); 2 Université de Lorraine, CNRS, IMoPA, F-54000 Nancy, France; arnaud.bianchi@univ-lorraine.fr (A.B.); emilie.velot@univ-lorraine.fr (E.V.); 3 Université de Lorraine, Unité de Recherche Animal et Fonctionnalités des Produits Animaux (UR AFPA), 2 Avenue de la Forêt de Haye-BP 20163, F-54505 Vandœuvre-lès-Nancy, France; celine.cakir-kiefer@univ-lorraine.fr

**Keywords:** nanoliposome, marine peptide, polar lipid, drug delivery, LC-PUFA, byproduct

## Abstract

Salmon byproducts (*Salmo salar*) generated by the food chain represent a source of long-chain polyunsaturated fatty acids (eicosapentaenoic acid (EPA): 20:5*n*-3; docosahexaenoic acid (DHA): 22:6*n*-3) and peptides that can be used as supplements in food for nutraceutical or health applications, such as in the prevention of certain pathologies (e.g., Alzheimer’s and cardiovascular diseases). The extraction of polar lipids naturally rich in PUFAs by enzymatic processes without organic solvent (controlled by pH-Stat method), coupled with the production of 1 kDa salmon peptides by membrane filtration, allowed the formulation of nanocarriers. The physicochemical properties of the nanoliposomes (size ranging from 120 to 140 nm, PDI of 0.27, zeta potential between −32 and −46 mV and encapsulation efficiency) were measured, and the bioactivity of salmon hydrolysate peptides was assessed (antioxidant and antiradical activity: ABTS, ORAC, DPPH; iron metal chelation). Salmon peptides exhibited good angiotensin-conversion-enzyme (ACE) inhibition activity, with an IC_50_ value of 413.43 ± 13.12 µg/mL. Cytotoxicity, metabolic activity and proliferation experiments demonstrated the harmlessness of the nanostructures in these experimental conditions.

## 1. Introduction

The 2020 edition of The State of World Fisheries and Aquaculture continues to demonstrate the significant and growing role of fisheries and aquaculture in providing food and nutrition. Marine capture fisheries reached 81.2 million tons in 2018, and byproducts, which represent 40 to 60 percent of total fish weight, are usually composed of heads (9–12 percent), viscera (12–18 percent), skin (1–3 percent), bones (9–15 percent) and scales (about 5 percent) [1,2].

The current industrial challenge involves the second-degree valuation, which is the transformation into dietary, cosmetic and nutraceutical supplements. This involves the extraction of several valued biomolecules by hydrolysis, preferentially using “Generally Recognized As Safe” (GRAS) proteolytic enzymes: extraction of refined oils (vitamins A and D, alkylglycerols, omega 3 and 6 polyunsaturated fatty acids), protein hydrolysate and other various molecules (chitin, chitosan, collagen, keratin, gelatin, etc.) [3]. This potential reservoir of naturally occurring long-chain polyunsaturated fatty acids (LC-PUFAs) has therapeutic and preventive value, and most international health organizations recommend their consumption. Current recommendations for eicosapentaenoic acid (EPA; 20:5*n*-3) and docosahexaenoic acid (DHA; 22:6*n*-3) intake for general health vary from country to country but are typically 400 to 500 mg/day as a combination of both fatty acids. The intake of LC-PUFAs as dietary supplements appears to be a promising strategy in the prevention and management of sarcopenia, especially when combined with healthy dietary patterns and anabolic stimulus induced by physical activity [4]. Fish consumption or dietary supplements (minimum intake of 4.0–4.5 g daily of *n*-3 PUFAs) could be considered functional food for elderly people with sarcopenia [5]. PUFAs may also affect the balance of platelet aggregation in blood vessels [6] and could maintain normal brain function in elderly people with an Alzheimer pathology risk [7]. 

Furthermore, fish hydrolysates from these byproducts contain bioactive peptides. Depending on the amino acid sequences resulting from the activity of the protease used, which can release more or less hydrophobic terminal amino acids, peptides may possess various functions, such as antioxidant [8,9,10], antihypertensive [11], antimicrobial, antifungal [12], immunomodulatory, antiproliferative [13] or antidiabetic [14] activities.

The reported positive effects of hydrolysates of marine byproducts could be useful in the prevention of certain emerging diseases. Indeed, the regular consumption of food supplemented with these biofunctional molecules could help to reduce the risk related to disabling multifactorial pathologies (Alzheimer’s and cardiovascular diseases). The nature of such natural active biopeptides can easily be integrated into various food matrices, which could be an effective method of consumption. Moreover, biopeptide activity could be enhanced by using marine polar lipids naturally rich in LC-PUFAs (EPA and DHA) assembled in the form of nanoliposomes dispersed in aqueous solution. The latter has already showed interesting properties in the prevention of several diseases (Alzheimer’s, cancer, etc.).

This research work aims to illustrate the bioactive properties of hydrolysate fractions of salmon head proteins vectorized by liposomal matrices naturally rich in LC-PUFA polar lipids, derived from the same byproduct.

Cytocompatibility studies of these lipid vectors integrating a peptide fraction of 1 kDa obtained by ultrafiltration were carried out on primary human mesenchymal stem cells (MSCs) isolated from human Wharton’s jelly.

## 2. Results and Discussion

### 2.1. Amounts of Proteins and Peptides

In this study, salmon heads were hydrolyzed by Alcalase^®^ enzyme, as described by Gbogouri et al. [15], and defatted, and the aqueous fraction was recovered. From the latter, three fractions were obtained via ultrafiltration of the recovered aqueous fraction with three different ceramic membrane cut-off sizes (i.e., 0.1 µm pore size, 10 kDa and 1 kDa). The obtained permeates were freeze-dried and termed salmon head hydrolysate (SHH) and labeled as SHH1, SHH2 and SHH3 for 0.1 µm pore size, 10kDa and 1 kDa, respectively. The total protein and ash and the nitrogen composition of hydrolysate were studied, using the same amounts of product for each assay (Table 1 and Table 2). As shown in Table 1, the protein content of SHH1 was 47.34% and significantly increased (*p* ≤ 0.05) after using other cut-off size membranes (i.e., 10 kDa and 1 kDa). The amount of SHH2 and SHH3 were 59.07% and 60.90%, respectively. According to the results, SHH fractions may contain more than 30% mineral material. As seen in Huang et al. [16] and Pati et al. [17], fish hydrolysates can contain abundant amounts of minerals, especially when the byproduct used contains scales. This result indicates that SHH fractions are rich in amino acids, peptides and proteins linked to other components. It is important to indicate that the process used made it possible to obtain a complex hydrolysate, which thus may contain several types of molecules and components.

In addition, the nature of the nitrogen composition for each permeate (Table 2) shows significate intraspecific differences. The total nitrogen value (N_T_) of SHH2 was significantly higher (*p* ≤ 0.05), with a value of 84.32%, than those of SHH1 and SHH3, which were 76.64% and 74.36%, respectively, indicating that SHH2 contains higher peptide content than SHH1 and SHH3. In contrast, the non-soluble protein (N_P_) was higher in SHH1 (with a value of 16.74%) than SHH2 and null in SHH3, linked to the decrease in the exclusion size of the membrane. Then, the soluble hydrolysate nitrogen (N_sH_), such as amino acids and peptides, was higher in SHH3 (with a value of 74.36%) than in SHH1 and equal to that in SHH2, which was also correlated with the decrease in the exclusion size of the membrane. The SHH3 N_sH_ value is considered the same as the N_T_ value according to Yvon et al. [18].

### 2.2. Analyses of Antiradical Activities

The notion of antiradical activity includes free radical scavenging activities, reactive oxygen scavenging (ROS) activity and the chelation of pro-oxidant metal ions. To define the antiradical scavenging capacity, it is necessary to multiply the analytical tests, since there is no standard method that can estimate all of these parameters [19]. In this study, the antiradical activity of SHHs was determined by measuring synthetic free radical electron trapping activity (2,2-azinobis(3-ethylbenzothiazoline-6-sulfonic) acid (ABTS) and 2,2-diphenyl-1-picrylhydrazyl (DPPH)) and the protective capacity through the oxygen radical absorbance capacity (ORAC) test, as well as the reduction of ferrous iron.

Antiradical activity against ABTS quantifies electron transfer (ET) activity [19]. In this case, the ABTS test was used to quantify the antiradical scavenging capacity in a water-soluble system. By reducing the preformed radical cation (ABTS+•), the reaction results in a decrease in the absorbance measured at 734 nm. The results are summarized in Table 3. The results obtained show that SHH2 was significantly more effective (*p* ≤ 0.05), with a half-maximal inhibitory concentration (IC_50_) value of 27.17 µg/mL and a Trolox equivalent antioxidant capacity (TEAC) of 186.65 µM Trolox equivalent (TE)/mg, than SHH1 and SHH3, according to the statistical treatment. Thus, it could have no significant impact in view of the supposed biological purpose. Regarding glutathione (GSH), SHH antiradical scavenging values were significantly lower (*p* ≤ 0.05). However, the IC_50_ ABTS scavenging and TEAC values of SHHs are significant since the quantities expressed by IC_50_ are low. These results suggest that salmon head treated with Alcalase^®^ led to the release of peptides with antiradical scavenging activity as measured by ABTS. Borawska et al. [9] showed that *Salmo salar* L. proteins digested by human gastric and duodenal juice using Corolase PP thoroughly break peptides bonds. The duodenal hydrolysate of salmon sarcoplasmic protein showed the highest ABTS scavenging value (72.7 ± 1.2%), which is linked to the release of high amounts of free amino acids relative to peptides after extended hydrolysis. Moreover, Sun et al. [20] investigated the antiradical scavenging activity of Alaska Pollock (*Theragra chalcogramma*) collagen hydrolysate using Alcalase^®^ and twice-simulated gastrointestinal digestion. Their ABTS scavenging results increased with increasing DH values. Furthermore, Tao et al. [21] were able to purify three antioxidant peptides prepared from spotless smooth-hound (*Mustelus griseus*) cartilage with various purification methods. The peptide sequences were Gly-Ala-Glu-Arg-Pro, Gly-Glu-Arg-Glu-Ala-Asn-Val-Met and Ala-Glu-Val-Gly. The peptides showed good half-maximal effective concentrations (EC_50_) with the ABTS method, i.e., 0.10, 0.05 and 0.07 mg/mL, respectively, which are comparable to our IC_50_ values obtained from the complex salmon head hydrolysate.

Antiradical activity against DPPH also quantifies electron transfer (ET) activity [19]. In addition to the ABTS method, the DPPH test was used to quantify the antiradical scavenging capacity in a lipophilic system. By reducing 2,2-diphenyl-1-picrylhydrazyl radicals (DPPH•), the reaction results in a decrease in the absorbance measured at 517 nm. The results are summarized in Table 3. The DPPH radical scavenging activity of SHH fractions was lower than the values obtained in the ABTS test, with IC_50_ values greater than 3 mg/mL and low TEAC equivalents compared to GSH values (IC_50_ 8.27 ± 0.71% and 718.36 ± 18.19 µM TE/mg). These results may be due to the loss of hydrophobic peptides and amino acids during the enzymatic hydrolysis, decreasing the potential for election quenching of DPPH [9], and the fact that we used only the recovered water-soluble fraction from the whole hydrolysate. As seen in the literature, salmon filet hydrolysates using various human digestive enzymes showed low DPPH scavenging activity (≈12% per mg), which increased when using the RP-HPLC purification process, which extracts bioactive compounds based on their hydrophobicity [10]. In addition, purified salmon sarcoplasmic and myofibrillar hydrolysates using human duodenal suc and Corolase PP showed a low DPPH scavenging value (8.88 ± 0.87% per mg) [9]. Furthermore, a small peptide fraction from salmon skin collagen hydrolyzed using purified bacterial extracellular proteases (Vibrio sp. SQS2-3) showed good DPPH quenching (73.29 ± 1.03%) due to their small size and hydrophobicity [8]. 

Oxygen radical absorbance capacity (ORAC) is a hydrogen atom transfer (HAT) test that involves peroxyl radicals generated using 2,2’-Azobis(2-amidinopropane) dihydrochloride (AAPH). The ability of the sample to protect fluorescein from oxidation makes it the standard typically used in this method [19], which can be used to investigate the absorption capability against peroxyl radicals in a sample. As shown in Table 3, the ORAC values of the SHH fractions ranged from 97.23 to 288.96 µM TE/mg sample, and there was a direct relationship with the cut-off size of the ultrafiltration membranes used. Clearly, the SHH2 and SHH3 fractions, which were obtained using ceramic membranes with 10 and 1 kDa cut-off sizes, showed high ORAC TE values, which are significantly higher in comparison to GSH (*p* ≤ 0.05). Glutathione (Glu-Cys-Gly) is known to be the most abundant low-molecular-weight thiol compound in cells, which plays a critical role in protecting cells from oxidative damage by interacting, reducing and conjugating with ROS compounds [22]. The results indicate that ultrafiltration fractions with a low cut-off size made SHH ROS quenching more effective. Girgih et al. [10] reported ORAC results ranging from 63 to 1541 µM TE/g peptide sample. The authors suggested that there is a direct relationship between the peptides’ hydrophobicity and the results obtained. Moreover, bacterial extracellular proteases were used to hydrolyze collagen and muscle proteins from salmon skin byproducts. The ORAC results of the fractionated hydrolysate showed good peroxyl radical quenching, with a value of 1.960 ± 0.381 mmol TE/g. However, the total oxygen absorbance capacity of our salmon head hydrolysate showed better results. Alcalase^®^ hydrolysate is a serine protease with low hydrolyzing specificity, with a preference for large uncharged amino residues [23] such as Trp, Cys and Tyr, which show radical scavenging activities that are enhanced when contained in peptides, especially when localized in N-terminus [24]. 

However, the chelation activity results remained good for SHH fractions. The results also show that reducing the cut-off ultrafiltration membrane size significantly decreased (*p* ≤ 0.05) the chelation activity. MCA activity has been observed in a multitude of peptides generated by marine products. Ben Khaled et al. [26] investigated the in vitro antioxidant activity of protein hydrolysates obtained from sardine muscle (*Sardinella aurita*) treated with different proteolytic enzymes. The results showed that up to 89% chelation activity could be obtained using 1 mg/mL sardine hydrolysate. Furthermore, Girgih et al. [10] reported MCA results of up to 34% per mg salmon protein hydrolysate. They suggested that inherent amino acids enhanced the iron-chelating activity and reduced it when separated from the whole fraction using RP-HPLC. The results were also linked to the amounts of glutamic acid and histidine, which give bioactive peptides more carboxylic groups and imidazole rings, enhancing the electrostatic interaction with Fe^2+^ ions. Moreover, protein hydrolysates obtained from cod (*Gadus morhua*) showed more than 80% Fe^2+^-chelating activity, with a higher activity attributed to a fraction size lower than 3kDa. The authors suggested that the amino acid composition of their fractions might have played an essential role in enhancing the metal chelation activity, particularly acidic and/or basic amino acids [27].

### 2.3. ACE Inhibition Activity Measurement of SHH3

**Figure 1:**
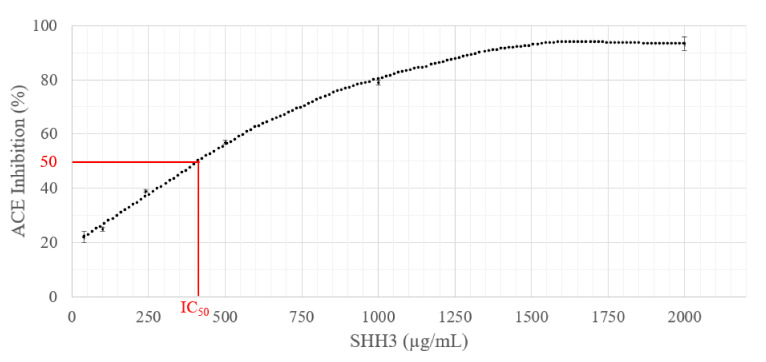
SHH3 ACE inhibitory value curve, ranging from 40 to 2000 µg/mL. IC_50_ (red line) indicate concentration peptide for half maximal ACE inhibition activity.

Among the SHH fractions obtained, only one was used to study potential antihypertensive activity in salmon head hydrolysate. The choice of the fraction to use was based on the molecular weight, taking into account that the most active peptides are small in size [23]. The ACE inhibitory activity of SHH3 was investigated, and the results are illustrated in Figure 1, which presents the drawn curve used for the estimation of the SHH3 IC_50_ value. SHH3 exhibited good ACE inhibition activity, with an IC_50_ value of 413.43 ± 13.12 µg/mL, showing that SHH3 contains bioactive peptides among the ultrafiltrated hydrolysate. Previous studies on the collagen hydrolysate of animal and fish byproducts using thermolysin showed that ACE inhibitory activity IC_50_ values ranged from 600 to 2800 µg/mL [28]. Furthermore, studies conducted on gelatin hydrolysates prepared from milkfish (*Chanos chanos*) scales showed that ACE inhibitory activity IC_50_ ranged from 547 to 762 µg/mL, whereas captopril exhibited a lower IC_50_ value of 0.002 µg/mL [16]. Captopril ACE inhibitory activity is still better than that of marine by-product hydrolysates. However, SHH3 showed better ACE inhibitory activity since the head contains several proteins (collagenous, myofibrillar and sarcoplasmic) if we take into account the flesh remaining on salmon head necks with more potent ACE inhibitory peptides containing uncharged tryptophan residues, which may contribute to the release of potent ACE inhibitory peptides [29].

### 2.4. Size, Polydispersity, Encapsulation Efficiency and Suspension Stability

The average particle size, polydispersity index, encapsulation efficiency and zeta potential of nanoliposomes encapsulating a concentration of 10 mg/mL salmon head hydrolysate fractions, SHH2 and SHH3, are summarized in Table 4. The data obtained were compared to empty nanoliposomes. The average size of the SHH nanoliposomes differed significantly (*p* ≤ 0.05) from that of the control, depending on the encapsulated fraction. The SHH2 fraction decreased the size from 118.93 ± 1.15 (control) to 53.91 ± 2.66 nm, whereas the SHH3 fraction increased the size to 143.87 ± 3.99 nm. Taking into account that all of the nanoliposomes were prepared in the same way, producing multilamellar nanoliposomes (MLVs), we suggest that the composition of SHH2 affects the average size of nanoliposomes since the cut-off size was higher than that of SHH3 (i.e., <10 kDa). SHH2 is rich in peptides and polypeptides with various molecular weights. The size of liposomes can vary according to many factors, such as the type, charge and concentration of the polymers and the technique used to produce them [30]. Da Rosa Zavareze et al. [31] were able to produce encapsulated protein hydrolysates from whitemouth croaker (*Micropogonias furnieri*) using Flavourzyme. The encapsulations were carried out through the lipid film hydration method using phosphatidylcholine from crude soy according to the classical method, where pure phospholipids dissolved in chloroform were evaporated in a rotary evaporator until the formation of a lipid film. The resulting lipid film was dispersed into buffer containing the lyophilized hydrolysate [31]. The average sizes were between 266 and 263 nm, while the control capsules were 208 nm. Moreover, Mosquera et al. [32] were able to design nanoliposomes using the sea bream scale hydrolysate peptide fraction (<3 kDa) and partially purified phosphatidylcholine via a film hydration method. The liposome size ranged from 66.2 to 214 nm. Furthermore, Hosseini et al. [33] fabricated nanoliposomes from fish skin gelatin hydrolysates using Alcalase^®^. 1,2-Dipalmitoyl-sn-glycero-3-phosphocholine (DPPC) and cholesterol were the main nanoliposome components, and the hydration film method was performed. The size range obtained was from 134 to 621 nm. However, none of them displayed a reduction in size. We suggest that the mass weight of the fraction and the method to produce the nanoliposomes were the main factors affecting the size.

The polydispersity indexes measured for the different nanoliposomes were higher than 0.2, so they can be regarded as polydisperse systems [31]. SHH2 nanoliposomes show a significantly different value from the others (i.e., control and SHH3 capsules) (*p* ≤ 0.05). The values indicate that control and SHH3 nanoliposomes are more stable than the SHH2 capsules. The changes in the size may increase Ostwald ripening and sedimentation in the system since the polydispersity index is high. The colloid stability was also evaluated through zeta potential measurements. Nearly all soluble particles acquire an electric surface charge when put into contact with water. This charge creates a repulsive force that keeps the colloid system stable and prevents aggregation. The results show that all nanoliposomes were negatively charged and slightly decreased when using encapsulated SHH fractions. The negative values recorded are due to polar phospholipids in marine lecithin [34]. The decrease in the negative surface charge of SHH nanoliposomes may be due to the high concentration of mineral compounds and/or peptides and amino acids that are positively charged. The encapsulation efficiency (EE%) represents, in this case, the capacity of the nanoliposomes formed to carry SHH compounds in their different compartments (i.e., core, lipid bilayer). From the data obtained, nanoliposomes encapsulating SHH fractions showed an EE% not exceeding 17%, which is normal when taking into account the size and hydrophilicity of the peptides and the nature of the phosphides [31,32,33,35].

### 2.5. Fourier Transform Infrared of Nanoliposomes

**Figure 2:**
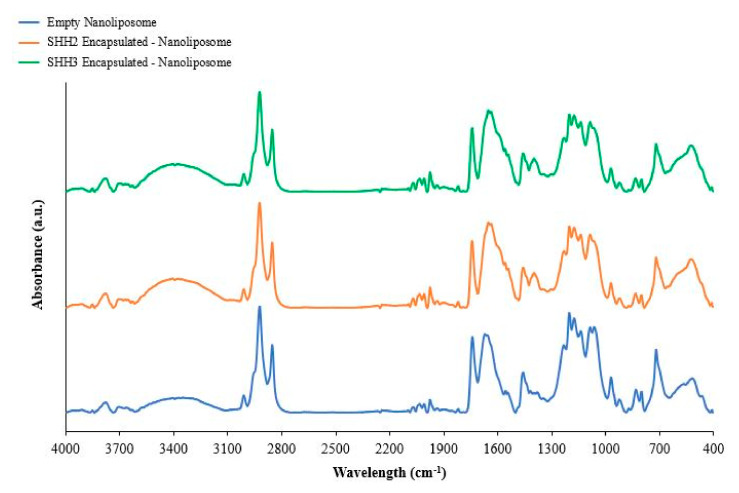
FTIR-ATR spectra of freeze-dried empty and SHH2- and SHH3-encapsulated nanoliposomes.

The nanoliposomes were analyzed by infrared spectroscopy in order to confirm the interaction between marine lecithin and the SHH fractions used to perform the encapsulation. FTIR spectra in the range 4000–400 cm^−1^ (Figure 2) were used to identify the changes that occur in the nanoliposome compartment by comparing each SHH nanoliposome with the control. The results of significant functional groups and their band shifts are presented in Table 5. 

Significant shifts were recorded for both nanoliposomes encapsulating SHH fractions compared to the control, with significant similarities and differences when comparing both. SHH2 modified the axial stretching of the carbonyl group (C=O) and the stretching vibration of alkene carbon–carbon (-C=C-) bonds, which shifted to higher frequencies, from 1648 to 1639 cm^−1^ and 1654 to 1645 cm^−1^, respectively, in nanoliposomes. SHH3 modified the C=O and -C=C- bonds in the nanoliposomes, but with a smaller shift, from 1648 to 1646 cm^−1^ and from 1654 to 1652 cm^−1^, respectively, on the basis of shifts detected in the asymmetric axial stretching of the phosphate group’s (PO_2_) double bond and PO_2_ broad stretch, from 1162 to 1160 cm^−1^ and from 1090 to 1087 cm^−1^. The –C=C– shift corresponds to a decrease in acyl chain mobility and an increase in the order of the bilayer, and the C=O shift was linked to interactions via hydrogen-bond formation between the carbonyl group and the active compounds, which, in this case, represent amino acids and peptides [33]. The frequency of PO_2_ stretching indicates the presence of hydrogen bonds between the functional group and the hydrogen atom from amino acids or peptides [31]. 

The SHH fraction did not modify the stretching of choline (CH_3_)_3_N, CH_2_ and CH_3_ hydrophobic fatty acid groups of the nanoliposome, which can be interpreted as the absence of the SHH compounds in the compartments of both internal and external surfaces of the liposome but also between the lipid bilayers. The encapsulation of highly hydrophobic compounds using the method described by the Biomolecular Engineering Laboratory is not effective with hydrophilic peptide fractions and was determined to have a maximum encapsulation efficiency of 17%. However, the goal was to integrate bioactive peptides into a nanoliposome, which was designed and biologically effective.

### 2.6. Biocompatibility of Nanostructures

**Figure 3:**
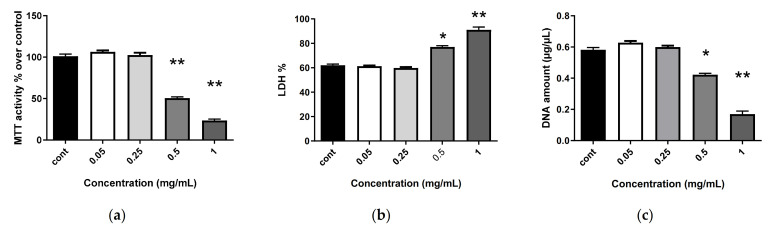
Impact of SHH3 on human MSC biocompatibility. Human MSCs were exposed to increasing concentrations of SHH3 (0.05, 0.25, 0.5 and 1 mg/mL) for 7 days. (**a**) Metabolic activity was assessed using the MTT assay. For each tested condition, the cell metabolic activity results are presented in percent versus control (cont) condition (100%); (**b**) LDH release was determined as described in the Materials and Methods section; (**c**) DNA concentrations were measured to estimate MSC proliferation. The results shown are mean ± SD of at least three individual experiments: * *p* < 0.01 and ** *p* < 0.001 compared to control condition for each time point.

**Figure 4:**
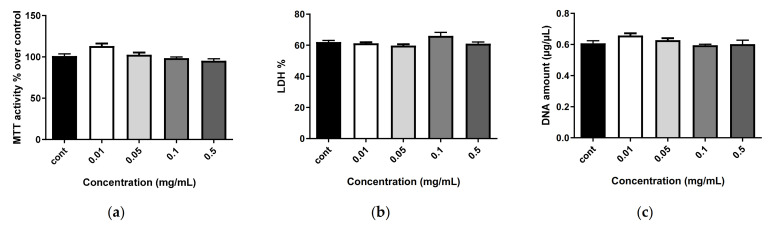
Impact of nanoliposomes on human MSC biocompatibility. Human MSCs were exposed to increasing concentrations of nanoliposomes (0.01, 0.05, 0.1 and 0.25 mg/mL) for 7 days. (**a**) Metabolic activity was assessed using the MTT assay. The cell metabolic activity results for the different membranes are presented in percent versus control results (as 100%); (**b**) LDH release was determined as described in the Materials and Methods section; (**c**) DNA concentrations were measured to estimate the proliferation of the cells. Statistical tests showed no significant difference between groups.

**Figure 5:**
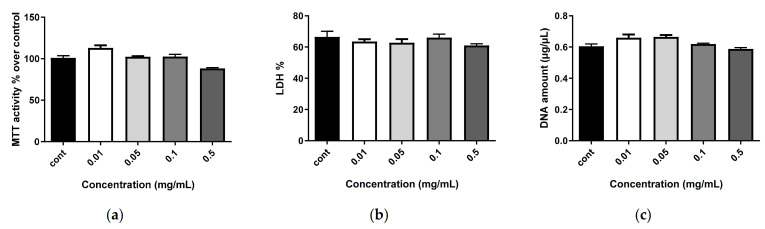
Impact of SHH3-encapsulated nanoliposomes on human MSC biocompatibility. Human MSCs were exposed to increasing concentrations of SHH3 and nanoliposomes (0.01, 0.05, 0.1 and 0.25 mg/mL of nanoliposomes and corresponding SHH3 concentrations of 0.25, 0.05, 0.025 and 0.005 mg/mL, respectively) for 7 days. (**a**) Metabolic activity was assessed using the MTT assay. The cell metabolic activity results on the different membranes are presented in percent versus control results (as 100%); (**b**) LDH release was determined as described under the Materials and Methods section; (**c**) DNA concentrations were measured to estimate proliferation of the cells. Statistical tests showed no significant difference between groups.

The data in Figure 3, Figure 4 and Figure 5 show the results of a 7-day treatment, as no large modifications were observed for shorter times (day 3 and day 5).

The evaluation of metabolic activity was based on the ability of living cells to reduce tetrazolium salt of MTT (3-(4,5-dimethylthiazol-2-yl)-2,5-diphenyltetrazolium bromide) into formazan crystals. All conditions showed important metabolic activity (Figure 3b), except for when exposed to the most elevated concentrations of free SH3 (500 and 1000 µg/mL). The cytotoxicity of SH3 was evaluated 1, 3, 5 and 7 days after exposure to various concentrations using the lactate dehydrogenase (LDH) assay (Figure 3a, only day 7 is shown). Cells not exposed to SH3 were used as the control. There was no statistically significant difference in cytotoxicity between the control and the following concentrations of SH3: 0.05 and 0.1 mg/mL. Among the different concentrations, only the highest ones, 0.5 and 1 mg/mL, appeared to be cytotoxic to human MSCs. Again, the two extreme concentrations triggered a decrease in proliferation, as assessed by DNA quantification (Figure 3c). 

The nanostructures alone (i.e., empty nanoliposomes (Figure 4, only day 7 is shown)) and nanoliposomes associated with SHH (Figure 5, only day 7 is shown) did not modify metabolic activity or proliferation and showed no cytotoxicity, regardless of the concentration (0.01, 0.05, 0.1 and 0.5 mg/mL) or the duration of stimulation (1, 3, 5 or 7 days). This demonstrates the harmlessness of the tested conditions.

## 3. Materials and Methods

### 3.1. Materials

Fresh salmon (*Salmo salar*) heads were obtained from a local plant and preserved at −20 °C. The substrate was thawed at 4 °C overnight before use. Alcalase^®^ 2.4 L (EC.3.4.21.14a; Novozymes A/S, Bagsvaerd, Danemark), sodium hydroxide (NaOH), hydrochloric acid (HCl), bicinchoninic acid kit, boric acid, sulfuric acid, 2,2-azinobis (3-ethylbenzothiazoline sulfonic acid) (ABTS), potassium persulfate (K_2_O_8_S_2_), phosphate buffer solution (PBS) made by using monobasic sodium (NaH_2_PO_4_) and dibasic sodium phosphate (Na_2_HPO_4_), glutathione (GSH), 6-hydroxy-2,5,7,8-tetramethylchroman-2-carboxylic acid (Trolox), fluorescein, 2,2′-Azobis(2-amidinopropane) dihydrochloride (AAPH), 2,2-diphenyl-1-picrylhydrazyl (DPPH), ferrous chloride (FeCl_2_), disulfonate monosodium salt hydrate (ferrozine), ethylenediaminetetraacetic acid (EDTA), hippuric acid, *N*-Hippuryl-His-Leu hydrate (HHL), 0.1 U angiotensin-converting enzyme from rabbit lungs (ACE), 2-(cyclohexylamino)ethanesulfonic acid (CHES), sodium chloride (NaCl), N-[(S)-3-mercapto-2-methylpropionyl]-l-proline (captopril) and trifluoroacetic acid (TFA) were purchased from Sigma-Aldrich^®^ (Merck KGaA, Darmstadt, Germany). Centrisart^®^ tubes with 100 kDa exclusion size (Sartorius^®^, Goettingen, Germany), trichloroacetic acid (TCA), Cu-Se catalyzer (Panreac Quimica Sau^®^, Castellar del Vallès, Spain) and methanol (BioSolve^®^, BioSolve Chimie, Dieuze, France) were also used.

### 3.2. Preparation of the Salmon Head Hydrolysate (SHH)

After enzymatic hydrolysis of the salmon heads, as previously described by Gbogouri et al. [15], the hydrolysate was partially defatted using an industrial cream separator, aliquoted in bottles (800 mL) and stored at −20 °C until use. The partially defatted hydrolysate was thawed and centrifuged at 9000 rpm for 20 min at 4 °C using a Beckman Coulter^®^ Rotor J-10 centrifuge (Beckman Coulter, Brea, CA, USA). This allows for initially obtaining the three following visible fractions: the oil supernatant, the aqueous phase containing the desired peptides, and a pellet containing the heaviest fraction. Recovery of the aqueous fraction was achieved through vacuum suction and first filtered through Whatman^®^ paper number 3 (Cytiva Europe GmbH, Velizy-Villacoublay, France). After recovering the fraction of study interest, it was treated with a tangential ultrafiltration system with a ceramic tubular membrane with porosity 0.1 μm, 10 kDa and 1 kDa permeability, with the inlet flow set at 500 mL/min and pressure maintained at 3 bar. The permeates were recovered, freeze-dried and stored at 20 °C until use. The three different powders obtained were used in further manipulations and are abbreviated SHHs (i.e., salmon head hydrolysate). SHH1, SHH2 and SHH3 denote the 0.1 µm, 10 kDa and 1 kDa permeate, respectively.

### 3.3. Determination of Characteristics of SHHs

#### 3.3.1. Amounts of Proteins and Peptides and Nature of Nitrogen Composition

The amounts of proteins and peptides and the nature of the nitrogen composition in the SHHs were quantified by two different methods. The first was the use of an acid bicinchoninic assay kit (BCA). The second was the Kjeldahl method using an automaton mineralizer and Gerhardt^®^ distiller (C. Gerhardt GmbH & Co. KG, Königswinter, Germany), the method having been adapted according to ISO 1871 [36] and Yvon et al. [18].

##### Protein Content Using BCA Kit Method

An amount of 100 μL of SHH fractions with a concentration of 0.66 mg/mL was dispensed in a test tube and supplemented with 2 mL of a solution composed of 98% of reagent A (bicinchoninic acid, sodium carbonate, sodium tartrate, sodium bicarbonate in 0.1 N NaOH) and 2% reagent B (4% copper sulfate pentahydrate). The mixture was homogenized and then incubated at 37 °C for 30 min. The absorbance reading was performed at a wavelength of 562 nm. A calibration curve (0–800 μg/mL) was carried out using the standard provided with the kit (1 mg/mL bovine serum albumin (BSA) in 0.15 M NaCl and 0.05% azide sodium). The protein concentration in the SHHs was calculated from the calibration curve obtained.

##### Protein Content Using Kjeldahl Method

The analysis was carried out on 40 mg of SHHs diluted in 4 mL of distilled water to quantify the amount of total nitrogen (N_T_), non-soluble protein and peptide nitrogen (N_P_), as well as the non-protein nitrogen contained in the supernatant, namely, amino acids, soluble peptide nitrogen (N_sH_). The analyses were performed on SHHs pretreated with TCA. A total of 40 mg of each SHH was diluted in 1.6 mL of 24% TCA, and the mixture was increased to a volume of 4 mL using 12% TCA and resuspended in 4 mL of distilled water. The samples were put into Kjeldahl tubes and were supplemented with 2 mL of sulfuric acid and a pinch of Cu-Se catalyzer, and then the tubes were placed in the mineralizer for 2 h at 400 °C. The amount of nitrogen contained in each sample was titrated using a Gerhardt VAPODEST^®^ distiller (C. Gerhardt GmbH & Co. KG, Königswinter, Germany).

#### 3.3.2. Thermogravimetric Analysis (TGA)

TGA of SHHs was carried out for a quantitative estimation of mineral composition [17] using a TGA5500 TA^®^ instrument. The temperature range was 30–600 °C with a step of 20 °C/min. Results were treated using TRIOS^®^ Software (TA Instrument, New Castle, DE, USA).

### 3.4. Bioactivity Assessment

#### 3.4.1. ABTS•+ Scavenging Activity Assay

Quantification of the ABTS•+ scavenging activity was adapted according to the method used in [9]. The preformed organic and cationic radical of 2,2-azinobis (3-ethylbenzothiazoline sulfonic acid) (ABTS•+) was generated by the oxidation of ABTS at 7 mM using potassium persulfate (K_2_O_8_S_2_) at 2.45 mM after incubation for 12–16 h at 20 °C and in the dark, giving the solution a characteristic green color. Once the stock solution was ready, it was diluted in sodium phosphate buffer (PBS) (5 mM, pH 7.4) and incubated for 1 h at 20 °C in the dark. It is important to measure the absorbance of the single radical obtained at a wavelength of 734 nm and obtain values between 0.65 and 0.70. Finally, 100 μL of sample at different concentrations diluted in PBS were mixed with 900 μL of ABTS• +, well shaken and left in the dark at 20 °C for 10 min. GSH at different concentrations was used as a comparative control. A calibration curve using Trolox at different concentrations (0–20 μM) was also generated. ABTS•+ scavenging activity values were then expressed as µM Trolox equivalent (TE) per milligram of sample. The absorbance measurement was made at 734 nm. ABTS•+ scavenging activity (%) values were estimated as follows:

(1) $$\mathrm{ABTS}\;\mathrm{radical}\;\mathrm{scavenging}\;\mathrm{activity}\;\left(\%\right)=\left(\frac{B-A}{B}\right)\times 100$$

where *A* is the absorbance of the ABTS•+ reaction with the different samples; *B* is the absorbance of the ABTS•+ reaction with the blank.

#### 3.4.2. Determination of Oxygen Radical Absorbance Capacity (ORAC)

The ORAC was adapted from Cao et al. [37] with slight modifications. The SHH fractions were dissolved in sodium phosphate buffer (PBS) (75 mM, pH 7.4) at different concentrations. The samples were mixed with 2 mL of fluorescein solution (26 nM), followed by incubation of the mixture in the dark at 37 °C for 10 min. Afterward, an aliquot of AAPH (664 mM) was added to the mixture. The fluorescence variation due to AAPH-induced oxidation of fluorescein was measured at 2 min intervals for 40 min at excitation and emission wavelengths of 490 nm and 524 nm, respectively. GSH at different concentrations was used as a comparative control. ORAC values were expressed as µM Trolox equivalent (TE) per milligram of sample after generating a calibration curve using Trolox at different concentrations (0–100 µM). Fluorescence measurements were normalized to the curve of the blank (PBS), and then the area under the fluorescence decay curve (AUC) was calculated as follows:

(2) $$\mathrm{AUC}=1+\sum_{i=2}^{i=40}\frac{f_{i}}{f_{0}}$$

where *f*_0_ is the initial fluorescence reading at 0 min; *f_i_* is the fluorescence reading at time *i*. The net AUC of the sample was calculated by subtracting the AUC of blank.

#### 3.4.3. DPPH Radical Scavenging Activity

Quantification of 2,2-diphenyl-1-picrylhydrazyl (DPPH) antiradical activity was adapted from Girgih et al. [10]. A 500 μL sample at a concentration of 1 mg/mL was added to 500 μL of DPPH at a concentration of 0.1 mM diluted in methanol. Homogenization was carried out using a vortex, then the sample was incubated in an oven in the dark at 30 °C for 30 min. Absorbance was measured at a wavelength of 517 nm. GSH at different concentrations was used as a comparative control. A Trolox calibration curve at different concentrations (0–350 μM) was also generated, which was used to express the results as µM Trolox equivalent (TE) per milligram of sample. The estimate of the DPPH scavenging activity was made as follows:

(3) $$\mathrm{DPPH}\;\mathrm{radical}\;\mathrm{scavenging}\;\mathrm{activity}\;\left(\%\right)=\left(\frac{B-A}{B}\right)\times 100$$

where *A* is the absorbance of the DPPH reaction with the different samples; *B* is the absorbance of the DPPH reaction with the blank.

#### 3.4.4. Iron Metal Chelation Activity (MCA)

The iron metal chelation activity was measured using the method developed by Canabady-Rochelle et al. [25], with some modifications. Iron ions that are not chelated by peptides form an iron–ferrozine complex that is measurable at a wavelength of 562 nm. The test was carried out with a volume of 555 μL of different sample concentrations, to which 15 μL of a 2 mM solution of ferrous chloride (FeCl_2_) was added. After stirring, the mixture was incubated for 3 min at room temperature before adding 50 μL of ferrozine to 5 mM. After stirring and 10 min incubation, the absorbance was measured at 562 nm. EDTA at different concentrations was used as a comparative control. The ability to chelate iron (II) ions was calculated as follows:

(4) $$\mathrm{Metal}\;\mathrm{chelation}\;\mathrm{activity}\;\left(\%\right)=\left(\frac{B-A}{B}\right)\times 100$$

where *A* is the absorbance of the MCA of the different samples; *B* is the absorbance of the MCA of the blank.

#### 3.4.5. Angiotensin-Converting Enzyme (ACE) Inhibitory Activity

To measure the enzymatic activity of ACE, a method adapted from Chushman and Cheung [38] was used. It entails measuring the hippuric acid released after the HHL hydrolysis by RP-HPLC. For this purpose, 20 µL of recombinant ACE was incubated with 120 µL of 5.2 mM HHL diluted in CHES buffer (50 mM CHES and 300 mM NaCl, pH 8.3) in addition to 50 µL of different concentrations of samples at 37 °C for 60 min. The enzymatic reaction was then stopped by adding 75 µL of a solution containing 15 μM captopril, 3 mM EDTA and 0.2% TFA. The samples were filtered (0.45 μm) and loaded (50 μL) on an Alltima^®^ C18 column (2.6 μm, 100 Å, 150 × 2.10 mm; Avantor Inc., Radnor, PA, USA). A linear gradient of 13 to 50% ACN in ultrapure water in the presence of 0.1% TFA was applied for 7 min at a flow rate of 0.25 mL/min at 29 °C. Detection of hippuric acid was recorded at 228 nm. Peak integration in arbitrary units (AU) was performed using the software LC-Solution^®^ (Shimadzu, Kyoto, Japan). A calibration curve was generated upstream by injecting increasing amounts of hippuric acid (0–12 nM).

### 3.5. Encapsulation and Characterization of SHH Nanoliposomes

#### 3.5.1. Encapsulation of SHH Fractions

The preparation of the different multilamellar nanoliposomes was carried out according to the method of Bouarab et al. [34] with some modifications. First, 30 mg of each SHH fraction (SHH2 and SHH3) was added to 2.94 mL of ultrapure water and then mixed with 60 mg of marine lecithin to obtain a 2% dispersion of marine lecithin (*w/w*). The suspensions were stirred for 4 h. A fourth nanoliposome formulation (i.e., without any SHH fraction) was used as an empty control liposomal dispersion. Once the time elapsed, the dispersion was subjected to sonication (Sonicator Vibra-Cell 75115), with the parameters set at 30% intensity for 4 min (1 s on and 1 s off). The temperature was controlled using an ice water bath. An aliquot of each of these nanoliposomes was harvested and freeze-dried for Fourier transform infrared spectroscopy (FTIR) analysis.

#### 3.5.2. Characterization of SHH Fraction-Encapsulated Nanoliposomes

##### Encapsulation Efficiency

The encapsulation efficiency (EE) of the SHH peptides in the nanoliposomes was determined using the indirect method described by da Rosa Zavareze et al. [31], with some modifications. A quantity of 25 μL of each nanoliposome (Lip lecithin 2% 1 kDa or 10 kDa 10 mg/mL) was diluted in a volume of 975 μL of ultrapure water and then placed in Centrisart^®^ tubes with 100 kDa exclusion size (Sartorius^®^, Goettingen, Germany), which can retain the nanoliposomes and let the unencapsulated SHH pass. The parameters of the centrifugation were as follows: Beckman Coulter^®^ Rotor J-20 (Beckman Coulter, Brea, CA, USA) at 8000 rpm for 20 min at 20 °C. The quantity of non-encapsulated proteins was measured using the bicinchoninic acid (BCA) acid kit and expressed in μg eq. BSA/mL. The encapsulation efficiencies were estimated as follows:

(5) $$\mathrm{Encapsulation}\;\mathrm{Efficiency}\;\left(\%\right)=100-\left(\frac{A}{B}\times 100\right)$$

where *A* is the equivalent in µg BSA/mL of the non-encapsulated SHH; *B* is the equivalent in µg BSA/mL of the different SHH fractions, SHH2 and SHH3.

##### Dynamic Light Scattering and Zeta Potential Measurements

The size of the liposomes and their zeta potential were measured through dynamic light scattering (DLS) and electrophoretic light scattering (ELS) using a Zetasizer Nano ZS from Malvern Instruments^®^ (Malvern, UK). The protocol was adapted from Hasan et al. [39]. Empty liposome and SHH2 and SHH3 liposome samples were diluted to one-five-hundredth (1:500) in ultrapure water and then were spotted with a syringe into a zeta cell. An algorithm built into the Malvern^®^ Zetasizer software (Malvern Instruments, Malvern, UK) analyzed the measured intensity autocorrelation functions. All measurements were made at 25 °C.

##### Fourier Transform Infrared (FTIR) Spectroscopy

Fourier transform infrared spectroscopy was performed on empty and charged (i.e., SHH2 and SHH3 10 mg/mL) freeze-dried liposomes for qualitative comparison using a Bruker^®^ Tensor infrared spectrometer 27 (Bruker, Billerica, MA, USA) coupled with attenuated total reflectance (ATR). The protocol was adapted according to Mohan et al. [40]. Infrared spectra were obtained as the average of triplicates (16 sweeps each) between 4000 and 400 cm^−1^ with a resolution of 2 cm^−1^. The results were processed using the OPUS^®^ software (Bruker Optik GmbH, Ettlingen, Germany).

### 3.6. Isolation and Culture of Human MSCs

Umbilical cords were collected at the Maternity Hospital of Nancy after obtention of a consent form signed by the pregnant mothers in compliance with the French national legislation regarding human sample collection, manipulation and personal data protection. This collection was approved by the Nancy Hospital Ethics Committee and French Ministry of Higher Education, Research and Innovation (registration number no. DC2014-2114). Human umbilical cords (~20 cm) were obtained from full-term births (*n* = 3). Human Wharton’s jelly MSCs (MSCs) were isolated and expanded in culture according to standard protocols. Briefly, umbilical cord vessels were removed from cord segments, and exposed mesenchymal tissue was cut into small pieces (1–2 mm^3^) that were treated for 18 h with collagenase type 2 (1 IU/mL) (ThermoFisher, Walthma, USA) in alpha Minimal Eagle Medium (α-MEM) (Lonza, Basel, Switzerland) at 37 °C with 5% CO_2_ and 95% humidity. After centrifugation for 10 min at 300 g, MSCs were suspended and maintained in α-MEM supplemented with l-glutamine (2 mM), penicillin (100 U/mL), streptomycin (100 µg/mL) and 10% heat-inactivated fetal calf serum (FCS, Life Technologies, Carlsbad, CA, USA) in a 95% humidified atmosphere of 5% CO_2_ at 37 °C with medium change every 2 days. All experiments were performed with cells after no more than 4 passages.

MSCs were incubated in the presence or absence of SH3 at 0.05, 0.25, 0.5 and 1 mg/mL and nanostructures (i.e., empty nanoliposomes or nanoliposomes associated with SH3) at 0.01, 0.05, 0.1 and 0.5 mg/mL for 3, 5 or 7 days.

### 3.7. Biocompatibility Assays

To evaluate the impact of the various systems (SHH, nanoliposomes alone and SHH-encapsulated nanoliposomes) on cell behavior, different parameters were estimated: system potential cytotoxicity, cell metabolic activity and cell proliferation.

#### 3.7.1. Cytotoxicity Assay

The cytotoxicity test was performed after 3, 5 and 7 days using the Cytotoxicity Detection KitPLUS (LDH) (#04744926001; Roche, France) according to the manufacturer’s instructions. This assay is based on the measurement of lactate dehydrogenase (LDH) activity released from the cytosol of damaged cells. Three controls were included: background control (assay medium), low control (untreated cells corresponding to the control condition) and high control (a positive control where a maximum of LDH is released due to cell lysis). The absorbance was read on a spectrophotometer at 490 nm (Varioskan^®^ Flash, Thermo Scientific, Illkirch Graffenstaden, France). To determine the experimental absorbance values, the average absorbance values of triplicate samples and controls were calculated and subtracted from the absorbance values of the background control. The percentage of cytotoxicity was determined relative to the value of the high control (fixed to 100%).

#### 3.7.2. Cell Proliferation

Cell proliferation was assessed after 3, 5 and 7 days of MSC culture using Hoechst assay, which allows cell DNA quantification, as previously described [41]. Briefly, MSCs were harvested from 12-well plates and suspended in 100 µL of Hoechst buffer (10 mM TRIS, 1 mM EDTA and 0.1 M of NaCl, pH 7.4) before 5 series of freezing (liquid nitrogen)/thawing (60 °C, 5 min) cycles to lyse cells and release their DNA into solution. Black flat-bottom plates with low fluorescent background were used to perform the assay, and a calf thymus DNA standard curve was used for the quantification. The samples were mixed with 2 µL of Hoechst solution (0.1 µg/mL final concentration), and the measurements of DNA samples and standards were performed by fluorescence spectrophotometry (360 nm excitation/460 nm emission, Varioskan^®^ Flash, Thermo Scientific, Illkirch Graffenstaden, France). The DNA concentration (µg/mL) of each sample was based on its fluorescence measurement relative to the standard curve. 

#### 3.7.3. Cell Metabolic Activity

Cell metabolic activity was measured using MTT ((3-(4,5-dimethylthiazol-2-yl)-2,5-diphenyltetrazolium bromide) assay as described elsewhere [41]. A volume of 50 µL of MTT solution was added to 200 µL of cell culture medium. Briefly, MSCs were incubated for 4 h (5% CO_2_, 95% humidity at 37 °C) to allow the yellow dye to be transformed into blue formazan crystals by mitochondrial dehydrogenases. The supernatant was removed, and this insoluble product was protected from light, dissolved by adding 200 µL of DMSO and gently mixed at 37 °C for 5 min. The supernatants were removed, protected from light and centrifuged, and their absorbance was read within 30 min using a Varioskan^®^ Flash (Thermo Scientific, Illkirch Graffenstaden, France) at 540 nm. The control condition for MSC metabolic activity was used as the reference value.

### 3.8. Statistical Analysis

Results are expressed as mean ± SD. Statistical analyses were performed with GraphPad Prism 6 (GraphPad Software, San Diego, CA, USA) using one-way ANOVA multiple comparisons followed by Tukey honest significant difference correction. Results with *p*-values of 5%, 1% or 0.1%, depending on the analysis, were considered significant.

## 4. Conclusions

This work proposes the valorization of salmon byproducts to produce nanoliposomes composed of LC-PUFAs encapsulating peptides obtained from the same source using a hydrolysis method. The results show that the peptide fractions have promising bioactivities, such as antioxidant and antihypertensive bioactivities. Moreover, except at extreme concentrations (0.5 and 1 mg/mL), SHH fractions did not negatively affect human MSC viability or integrity. In the same way, in our experimental conditions, we demonstrated that the nanoliposomes, as well as the combined nanoliposomes and SHH fraction structures, did not induce cytotoxicity or cell death in human MSCs, nor did they disrupt proliferation throughout the entire experimental period at any of the tested concentrations.

These in vitro experiments strongly suggest that salmon lecithin nanoliposomes and SHH fractions could be used to enhance nutrition to prevent some pathologies.

## Figures and Tables

**Table 1:** Protein and ash content (percent) of SHH fractions using BCA kit assay and TGA instrument.

Sample	SHH1	SHH2	SHH3
Protein (%)	47.34 ± 0.93 ^2^	59.07 ± 1.85 ^1^	60.90 ± 1.52 ^2^
Ashes (%)	30.85 ± 0.35	33.32 ± 0.94	32.86 ± 0.29

^1,2^ Values with different numbers indicate significant differences at the same concentration (*p* ≤ 0.05).

**Table 2:** Estimation of total nitrogen (NT), non-soluble protein nitrogen (NP) and soluble hydrolysate nitrogen (NsH) content (percent) using Kjeldahl method.

NT (%)			NP (%)			NsH (%)		
**SHH1**	**SHH2**	**SHH3**	**SHH1**	**SHH2**	**SHH3**	**SHH1**	**SHH2**	**SHH3**
76.64 ± 0.372 ^2^	84.32 ± 1.181 ^1^	74.36 ± 0.133 ^3^	16.74 ± 0.511 ^1^	11.95 ± 3.312 ^2^	-	59.90 ± 0.222 ^2^	72.38 ± 2.751 ^1^	74.36 ± 0.133

^1–3^ Values in the same assay section with different numbers are significantly different at the same concentration (*p ≤* 0.05).

**Table 3:** Radical scavenging (ABTS, DPPH and ORAC) and MCA activities of salmon head hydrolysates.

**ABTS**								**DPPH**							
**IC_50_ (µg/mL)**				**TEAC (µM TE/mg)**				**IC_50_ (µg/mL)**				**TEAC (µM TE/mg)**			
**SHH1**	**SHH2**	**SHH3**	**GSH**	**SHH1**	**SHH2**	**SHH3**	**GSH**	**SHH1**	**SHH2**	**SHH3**	**GSH**	**SHH1**	**SHH2**	**SHH3**	**GSH**
39.09 ± 3.52 ^3^	27.17 ± 1.17 ^2^	42.32 ± 1.35 ^3^	1.78 ± 0.08 ^1^	178.94 ± 3.52 ^2^	186.65 ± 0.65 ^2^	160.78 ± 2.41 ^2^	3928.35 ± 57.64 ^1^	3383.02 ± 144.16 ^2^	4537.03 ± 291.35 ^3^	3217.80 ± 100.48 ^2^	8.27 ± 0.71 ^1^	2.84 ± 0.05 ^2^	2.55 ± 0.07 ^2^	2.80 ± 0.04 ^2^	718.36 ± 18.19 ^1^
**MCA**								**ORAC**							
**IC_50_ (µg/mL)**								**TEAC (µM TE/mg)**							
**SHH1**		**SHH2**		**SHH3**		**EDTA**		**SHH1**		**SHH2**		**SHH3**		**GSH**	
238.71 ± 3.90 ^2^		265.26 ± 5.39 ^3^		301.73 ± 2.28 ^4^		20.49 ± 0.16 ^1^		97.23 ± 3.64 ^2^		288.96 ± 37.84 ^1^		254.18 ± 3.11 ^1^		143.10 ± 7.33 ^2^	

^1–4^ Values in the same assay section with different numbers are significantly different at the same concentration (*p ≤* 0.05). Abbreviations: salmon head hydrolysate (SHH); (2,2-azinobis(3-ethylbenzothiazoline-6-sulfonic) acid (ABTS); 2,2-diphenyl-1-picrylhydrazyl (DPPH); oxygen radical absorbance capacity (ORAC); metal chelation activity (MCA); Trolox equivalent antioxidant capacity (TEAC); half-maximal inhibitory concentration (IC_50_). The metal chelation activity assay (MCA) is an antiradical test that involves the chelation of ferrous metal ions (Fe^2+^). Samples that can capture Fe^2+^ ions act as antiradical compounds since the Fenton reaction is responsible for hydroxyl radical formation and radical chain reactions [25]. As shown in Table 3, the MCA results of SHH fractions are expressed in IC_50_ µg/mL and compared with EDTA results. The SHH results ranged from 238.71 to 301.73 µg/mL, showing low chelation activity compared to EDTA.

**Table 4:** Average particle size, polydispersity, encapsulation efficiency and suspension stability, as acquired by zeta potential of the nanoliposomes obtained from salmon head hydrolysates.

Sample	Average Size (nm)	Polydispersity Index	Encapsulation Efficiency (%)	Zeta Potential (mV)
Control empty nanoliposomes	118.93 ± 1.15 ^2^	0.28 ± 0.01 ^2^	-	−46.97 ± 1.10 ^1^
SHH2 nanoliposomes	53.91 ± 2.66 ^4^	0.34 ± 0.01 ^3^	13.77 ± 2.50 ^1^	−38.57 ± 2.83 ^3^
SHH3 nanoliposomes	143.87 ± 3.99 ^1^	0.27 ± 0.02 ^2^	17.27 ± 3.08 ^1^	−32.30 ± 0.361 ^4^

Data in the same column labeled with different numbers are significantly different (*p ≤* 0.05).

**Table 5:** Wavenumber (cm^−1^) of spectra of encapsulated SHH by FTIR-ATR analysis.

Stretching	Control Empty Nanoliposomes	SHH2 Nanoliposomes	SHH3 Nanoliposomes
(CH_3_)_3_N	969	969	968
Broad stretch PO_2_	1090	1090	1087 *
PO_2_	1162	1162	1160 *
Phosphoester bond	1236	1233 *	1233 *
C=O	1648	1639 **	1646 *
CH_2_ (symmetric)	2852	2852	2852
CH_3_	2923	2923	2922
-C=C-	1654	1645 **	1652 *

*^,^** Data indicating significant differences in the same line with a measurement resolution of 2 cm^−1^ (* *p* < 0.05, ** *p* < 0.01).
